# Supplementary material for: Population structure and diversification of Gymnospermium kiangnanense, a plant species with extremely small populations endemic to eastern China
Source: PeerJ. 2024 Jun 24;12:e17554. doi: 10.7717/peerj.17554 (PMC11210486; doi:10.7717/peerj.17554)
Supplement: Supplemental Information 1 [file peerj-12-17554-s001.docx]

**Table S1** Information of 21 pairs of polymorphic SSR primers for *G. kiangnanense* in this study.

| Primer No. | Primer sequence (5′–3′) | Repeat motif | Allele size rang (bp) | | Tm (°C) |
| --- | --- | --- | --- | --- | --- |
| 1 | F: GCTTTGAGCTTTGTGGGTTT | AAAG | | 164-184 | 57.5°C |
|  | R: CACCGCAAACCCAATCACAG |  | |  |  |
| 2 | F: ATTGGACTGGGACGAACCAC | AAG | | 94-100 | 57.5°C |
|  | R: AAGCAAAGCCGAGACAGCAA |  | |  |  |
| 3 | F: TCGTTCTGTTTTGGTAGGGA | AGA | | 196-202 | 57.5°C |
|  | R: GGGTCTCTGTGGGAGTCTCA |  | |  |  |
| 4 | F: CCAAAACCAAACGTGCACCA | AGG | | 174-177 | 57.5°C |
|  | R: TGCTTCCACCGGAGTTGAAT |  | |  |  |
| 5 | F: AGCTCTTGGTGTAGCCCTTG | ATG | | 197-200 | 57.5°C |
|  | R: CCAAGGCTCACTCAACAGGA |  | |  |  |
| 6 | F: ACATTGCAGCTCTACCCACA | CAC | | 176-188 | 57.2°C |
|  | R: TGTCCAGGAAACGTCACCTC |  | |  |  |
| 7 | F: ACCATCTCAGAGCCAACAGC | CAG | | 116-131 | 57.2°C |
|  | R: AGATGGTTCGGTGTGCCTTT |  | |  |  |
| 8 | F: GCTTCTCGGAAAACCAAGCA | CCA | | 181-190 | 55.6°C |
|  | R: TACCGACACGTGTCATCCAC |  | |  |  |
| 9 | F: GCCAATTTTGCAAGCTTCGC | CTA | | 162-168 | 55.6°C |
|  | R: CTCGTCGGTGCCACAGAAT |  | |  |  |
| 10 | F: TTTAGGTGCTGGAGCTGCTC | CTT | | 107-113 | 57.5°C |
|  | R: TGGGTTGTTTTGAGATCTGGGT |  | |  |  |
| 11 | F: TGTTCGTGGCGAAGATGGAA | GAA | | 135-138 | 55.6°C |
|  | R: GTCGGCACCCCTTTAACAGA |  | |  |  |
| 12 | F: CTCACAACCTGGACCACCTG | GAT | | 132-135 | 57.5°C |
|  | R: GGCTCTTCTTGGCGTCCTAG |  | |  |  |
| 13 | F: TGATGTTGCTGTTGCTGCTG | GCT | | 164-179 | 59.6°C |
|  | R: CCAACCCCGCGTAAAACCTA |  | |  |  |
| 14 | F: CAGCCGATTGAGCAAACAGG | GGT | | 188-191 | 57.5°C |
|  | R: GCCGGTCTCCAAACGATGAA |  | |  |  |
| 15 | F: CCTCCAAGTCTGATGGATCCA | GTA | | 179-185 | 57.5°C |
|  | R: TTGCTGCTGCTGAGACTTGA |  | |  |  |
| 16 | F: AACCATGTTGCTGGTGCTTG | TAG | | 109-118 | 57.5°C |
|  | R: CTGATGGCGATGGTGATGGT |  | |  |  |
| 17 | F: TGCTGAAAATGGTAGTGGTGGA | TCA | | 119-131 | 57.5°C |
|  | R: GTAACCAACCCAACCTCGCT |  | |  |  |
| 18 | F: GCGGAAGTGATGAACATGCC | TCC | | 183-187 | 57.5°C |
|  | R: GAGGCGGAGGATGATCCAAA |  | |  |  |
| 19 | F: TCCAAGCTCCCCATTGTTCC | TGA | | 189-192 | 57.5°C |
|  | R: ATCCCTGTTGGTGGTGGTTG |  | |  |  |
| 20 | F: TGCAAGTCCAACGCACTACA | TTC | | 192-195 | 57.5°C |
|  | R: TTCATCGGTCGGATCTGACC |  | |  |  |
| 21 | F: GCAGAAGCCACTAAGAAAGCA | TTC | | 148-157 | 57.5°C |
|  | R: CGGACGAAGATGGAAGAATGGA |  | |  |  |
