# Supplementary material for: Population structure and diversification of Gymnospermium kiangnanense, a plant species with extremely small populations endemic to eastern China
Source: PeerJ. 2024 Jun 24;12:e17554. doi: 10.7717/peerj.17554 (PMC11210486; doi:10.7717/peerj.17554)
Supplement: Supplemental Information 3 [file peerj-12-17554-s003.docx]

**Table S3** Summary of genetic statistics and Wright’s F-statistics of each locus in the eitht populations of *G. kiangnanense*.

| Locus | *N_A_* | *H_E_* | *H_O_* | *PIC* | *Fit* | *Fst* | *Fis* | *Nm* |
| --- | --- | --- | --- | --- | --- | --- | --- | --- |
| 1 | 3 | 0.068 | 0.056 | 0.066 | 0.198 | 0.117 | 0.092 | 1.889 |
| 2 | 3 | 0.367 | 0.070 | 0.304 | 0.826 | 0.513 | 0.643 | 0.237 |
| 3 | 2 | 0.014 | 0.014 | 0.014 | -0.006 | 0.044 | -0.053 | 5.429 |
| 4 | 2 | 0.007 | 0.007 | 0.007 | -0.003 | 0.022 | -0.026 | 11.143 |
| 5 | 2 | 0.081 | 0.056 | 0.077 | 0.177 | 0.080 | 0.106 | 2.888 |
| 6 | 5 | 0.507 | 0.189 | 0.468 | 0.662 | 0.585 | 0.185 | 0.178 |
| 7 | 6 | 0.762 | 0.413 | 0.721 | 0.476 | 0.540 | -0.141 | 0.213 |
| 8 | 3 | 0.075 | 0.035 | 0.073 | 0.455 | 0.056 | 0.423 | 4.243 |
| 9 | 3 | 0.531 | 0.378 | 0.447 | 0.299 | 0.296 | 0.003 | 0.594 |
| 10 | 3 | 0.450 | 0.084 | 0.404 | 0.828 | 0.780 | 0.220 | 0.071 |
| 11 | 2 | 0.007 | 0.007 | 0.007 | -0.006 | 0.044 | -0.053 | 5.429 |
| 12 | 2 | 0.028 | 0.028 | 0.027 | -0.011 | 0.021 | -0.033 | 11.491 |
| 13 | 4 | 0.291 | 0.175 | 0.275 | 0.395 | 0.318 | 0.113 | 0.536 |
| 14 | 2 | 0.160 | 0.175 | 0.147 | -0.136 | 0.168 | -0.366 | 1.234 |
| 15 | 3 | 0.485 | 0.385 | 0.427 | 0.270 | 0.355 | -0.132 | 0.455 |
| 16 | 3 | 0.586 | 0.979 | 0.500 | -0.657 | 0.088 | -0.816 | 2.606 |
| 17 | 3 | 0.509 | 0.923 | 0.389 | -0.736 | 0.109 | -0.949 | 2.037 |
| 18 | 3 | 0.257 | 0.294 | 0.232 | -0.208 | 0.236 | -0.581 | 0.810 |
| 19 | 2 | 0.106 | 0.098 | 0.100 | 0.087 | 0.210 | -0.156 | 0.941 |
| 20 | 2 | 0.074 | 0.007 | 0.071 | 0.866 | 0.170 | 0.839 | 1.218 |
| 21 | 3 | 0.247 | 0.077 | 0.218 | 0.615 | 0.719 | -0.373 | 0.098 |
| Mean | 2.90 | 0.267 | 0.212 | 0.236 | 0.209 | 0.261 | -0.050 | 2.559 |
