# Supplementary material for: Population structure and diversification of Gymnospermium kiangnanense, a plant species with extremely small populations endemic to eastern China
Source: PeerJ. 2024 Jun 24;12:e17554. doi: 10.7717/peerj.17554 (PMC11210486; doi:10.7717/peerj.17554)
Supplement: Supplemental Information 4 [file peerj-12-17554-s004.docx]

**Table S4** Prior and posterior distributions for each parameter of scenario 1 obtained using DIYABC analysis.

| Parameter | Priors^a^ | Mean | Median | Mode | q025 | q050 | q250 | q750 | q950 | q975 |
| --- | --- | --- | --- | --- | --- | --- | --- | --- | --- | --- |
| N1（ZJZ） | (0-5,000) | 1.43×10^3^ | 1.17×10^3^ | 7.94×10^2^ | 2.72×10^2^ | 3.70×10^2^ | 7.33×10^2^ | 1.87×10^3^ | 3.42×10^3^ | 4.02×10^3^ |
| N2（ZJH） | (0-3,000) | 8.84×10^2^ | 8.25×10^2^ | 8.98×10^2^ | 2.63×10^2^ | 3.20×10^2^ | 6.00×10^2^ | 1.09×10^3^ | 1.68×10^3^ | 2.06×10^3^ |
| N3（AH） | (0-2,500) | 7.04×10^2^ | 6.17×10^2^ | 3.80×10^2^ | 1.34×10^2^ | 1.95×10^2^ | 4.24×10^2^ | 8.94×10^2^ | 1.42×10^3^ | 1.78×10^3^ |
| t1 | (0-1,200) | 3.84×10^2^ | 3.52×10^2^ | 2.34×10^2^ | 5.86×10^1^ | 9.06×10^1^ | 2.18×10^2^ | 5.07×10^2^ | 8.25×10^2^ | 9.65×10^2^ |
| t2 | (0-2,000) | 9.77×10^2^ | 9.32×10^2^ | 5.95×10^2^ | 2.35×10^2^ | 3.02×10^2^ | 6.04×10^2^ | 1.30×10^3^ | 1.83×10^3^ | 1.90×10^3^ |
