# Supplementary figures and images for: Population structure and diversification of Gymnospermium kiangnanense, a plant species with extremely small populations endemic to eastern China
Source: PeerJ. 2024 Jun 24;12:e17554. doi: 10.7717/peerj.17554 (PMC11210486; doi:10.7717/peerj.17554)

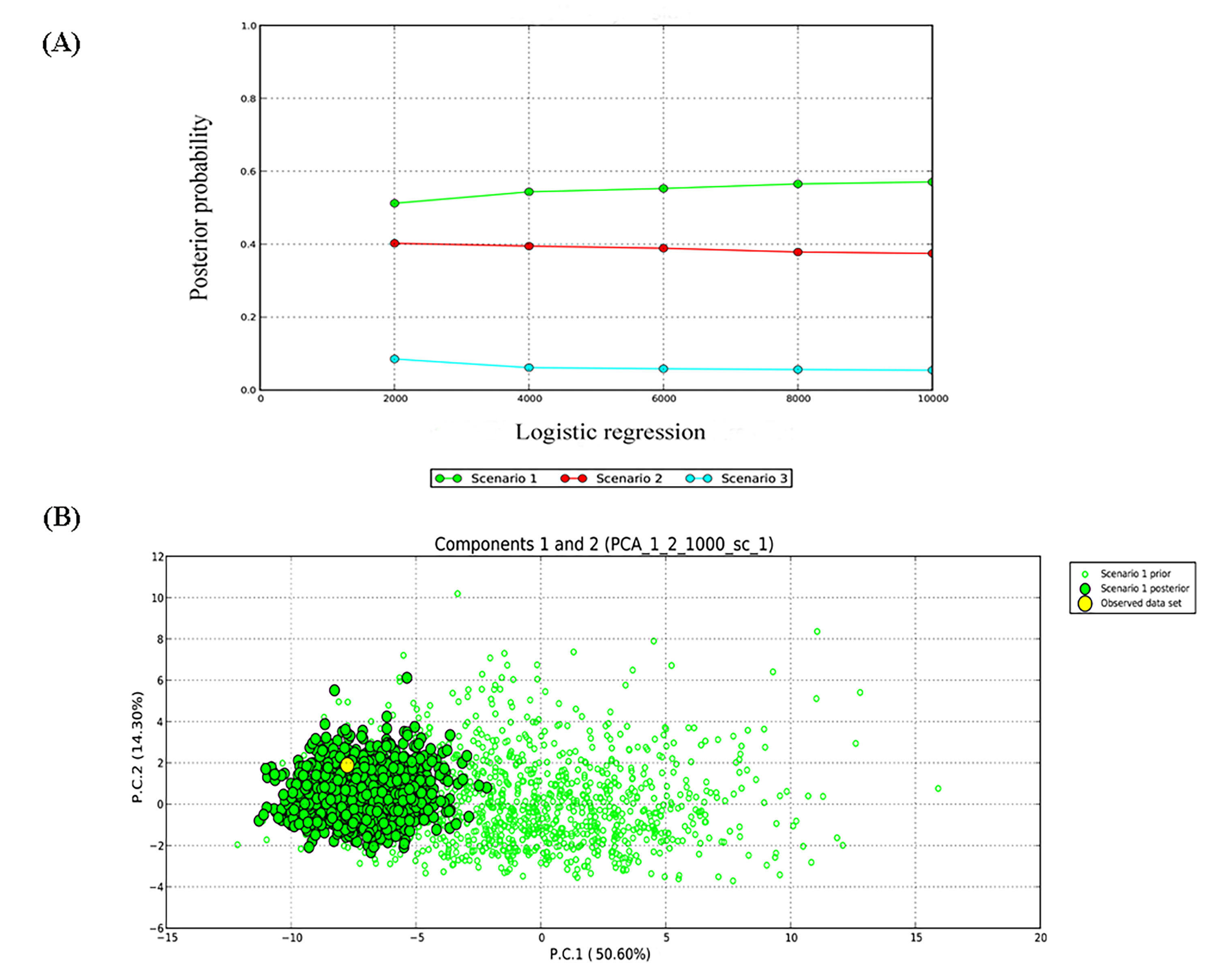

Supplement: Supplemental Information 5 — (A) Posterior probability of the three scenarios; (B) Goodness fit of Scenario 1 to the observed data evaluated in PCA analysis. [file peerj-12-17554-s005.png]
